# Beyond the Knife: Evolution, Innovation, Challenges, Global Gaps, and the Future of Acute Care Surgery

**DOI:** 10.7759/cureus.102209

**Published:** 2026-01-24

**Authors:** Olurotimi J Badero, Olutomiwa Omokore, Ibrahim O Quadri, Samuel O Ogunnoiki, Perelade Kingdom, Ogbuiyi-chima C Ifeanyichukwu, Temiloluwa Olayinka, Precious M Samuel-Ogunnoiki, Emmanuel S Meribole, Olaitan o Adeyoola, Nkechi Chima-Ogbuiyi, Bamikole Osibowale, Mariam O Buari, Julliete Umeh

**Affiliations:** 1 Interventional Cardiology, Iwosan Lagoon Hospital, Lagos, NGA; 2 Interventional Cardiology, Division of Cardio-Nephrology, Cardiac Renal and Vascular Associates, Jackson, USA; 3 Internal Medicine, Babcock University Teaching Hospital, Ilishan-Remo, NGA; 4 Internal Medicine, Benjamin S. Carson College of Health and Medical Sciences, Ilishan-Remo, NGA; 5 Surgery, Babcock University, Ilishan-Remo, NGA; 6 Surgery, Benjamin S. Carson College of Health and Medical Sciences, Ilishan-Remo, NGA; 7 Medicine and Surgery, Babcock University Teaching Hospital, Ilishan-Remo, NGA; 8 Surgery, General Hospital, Lagos, NGA; 9 Medicine and Surgery, Federal Medical Centre, Jabi, Abuja, NGA; 10 Medicine, Babcock University, Ilishan-Remo, NGA; 11 Nursing, Herzing University, Milwaukee, USA; 12 Cardiology, Carribean Tristate Heart Institute, Port of Spain, TTO; 13 Medicine, Babcock University Teaching Hospital, Ilishan-Remo, NGA; 14 Surgery, Igbinedion University, Okada, NGA

**Keywords:** acute care surgery, critical care innovation, ethical challenges, global disparities, multidisciplinary integration

## Abstract

Acute care surgery (ACS) is an integrated specialty encompassing trauma, emergency general surgery, and surgical critical care, aimed at delivering comprehensive, timely management of surgical emergencies. This narrative review examines the evolution of ACS from a procedure-focused model to a comprehensive perioperative care specialty. It analyzes significant innovations, including the use of artificial intelligence (AI) for sepsis prediction and complication risk stratification, the emerging role of robotic surgery, the incorporation of telemedicine, and the implementation of Enhanced Recovery After Surgery (ERAS) protocols. The review also identifies critical systemic challenges, including surgeon burnout, ethical dilemmas in end-of-life care, and chronic underfunding. Furthermore, it highlights substantial global disparities in the implementation of ACS models, with well-established systems in North America and fragmented or absent services in low-resource settings. This synthesis concludes that the future of ACS hinges on adopting technological advancements, addressing workforce sustainability, and developing policies to ensure equitable, high-quality emergency surgical care worldwide.

## Introduction and background

Acute care surgery (ACS) is commonly defined as a surgical specialty that integrates trauma surgery, emergency general surgery, and surgical critical care. It was developed to unify the expertise of trauma surgeons, emergency surgeons, and intensivists into a single, versatile, and comprehensive field [1]. It is also a dedicated surgical service designed to rapidly evaluate and manage patients with general surgical emergencies [2]. This field includes the treatment of injuries and conditions such as abdominal emergencies, acute appendicitis, and organ obstruction, among others. ACS also involves the delivery of preoperative and postoperative care and the management of complications that may arise before or after emergency surgeries [3].

Surgical critical care refers to the specialized care of patients who require intensive monitoring and management for severe or life-threatening conditions following surgery or trauma. It involves the use of advanced perioperative monitoring tools and techniques, both invasive and noninvasive, to assess and manage multiple physiological systems, including the cardiovascular, respiratory, and metabolic systems. The primary aim of this care is to optimize surgical outcomes by preventing, identifying, and treating postoperative complications, while also ensuring patient safety during the perioperative period. It also encompasses the use of technologies such as ultrasound, mechanical ventilation, and extracorporeal support systems to improve clinical outcomes and guide therapeutic interventions [4]. This article aims to review the evolution, key innovations, challenges, global gaps, and future directions of ACS as a surgical specialty.

## Review

Methodology

This study adopted a structured narrative review methodology to examine the evolution, innovations, challenges, global gaps, and future directions of ACS as a surgical specialty. A comprehensive literature search was conducted using PubMed, Google Scholar, and MEDLINE to identify relevant peer-reviewed articles, reviews, consensus statements, and guidelines related to ACS, trauma surgery, emergency general surgery, and surgical critical care. Search terms included “acute care surgery,” “trauma surgery,” “emergency general surgery,” “surgical critical care,” “critical care innovations,” “global surgery,” and “workforce models.” Only English-language articles with full-text availability were included, encompassing clinical studies, reviews, and policy- or systems-based publications. Extracted data focused on the historical development of ACS, models of care delivery, technological and critical care innovations, outcome measures, and disparities in global implementation. The findings were analyzed thematically and synthesized to provide a coherent overview of the current state and future trajectory of ACS, with all sources cited using the Vancouver referencing style to ensure accuracy and consistency.

Acute care surgery - importance of integrating emergency general surgery, critical care, and trauma management 

**Figure 1:**
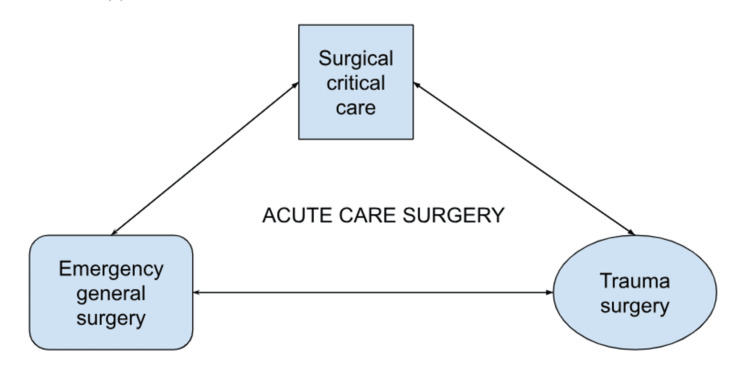
Chart describing the components of ACS and their interdependence ACS: acute care surgery

The impact of ACS in surgical care is important because of the cumulative amount of emergency and trauma surgery-related morbidity and mortality. The integration of emergency surgery, trauma care, and critical care has become increasingly essential in providing comprehensive patient management. As trauma and emergency surgical care evolve, it is no longer confined to general and trauma surgeons but has expanded to include various surgical specialties, each contributing unique expertise in managing trauma patients. This multidisciplinary approach ensures that trauma patients receive the most specialized care tailored to their needs [5]. This integration is crucial for managing critically ill patients, where surgical intervention may be followed by intensive critical care to address ongoing complications, and vice versa. A unified, multidisciplinary approach optimizes patient care by ensuring that all aspects of the patient's condition are addressed in a coordinated fashion, leading to reduced mortality rates and improved recovery times [5]. Figure 1 shows the interdependence and relationship between surgical critical care, emergency general surgery, and trauma surgery within the ACS specialty [1].

Moreover, the creation of the ACS specialty also enables better documentation and billing practices, underscoring the importance of accurate critical care time documentation for financial sustainability, as documented by Zolin et al. By focusing on a team-based (ACS team) approach, hospitals can optimize resource use, reduce mortality rates, and enhance the overall quality of emergency surgical and trauma care rather than involving multiple medical specialties at once [6].

Also, acute surgical admission wards (ASUs) offer a solution by providing short-term observation for patients needing close monitoring. This model helps reduce ER congestion and ensures that acute care surgeons skilled in both surgery and critical care manage patients more effectively. By integrating surgery, critical care, and trauma management, ASUs improve patient outcomes, streamline care, and enhance the efficiency of emergency surgical treatment [7]. 

The evolution of ACS and SCC: moving "beyond the knife" to comprehensive perioperative and critical care 

Acute care surgeons are general surgeons with additional board certification in surgical critical care who practice trauma, surgical critical care, emergency, and elective general surgery [8]. ACS was introduced as a new specialty by the American Association for the Surgery of Trauma in 2005 [9].

The concept of ACS developed in response to significant changes in trauma care patterns and increasing demands for specialized emergency surgical services. Over recent decades, the field of trauma surgery has undergone a substantial transformation. The incidence of penetrating trauma has decreased markedly, while many blunt trauma cases now require fewer surgical interventions due to advances in nonoperative management techniques such as improved imaging technology and the growing role of interventional radiology. At the same time, general surgery has become increasingly subspecialized, with many surgeons focusing on specific elective areas like bariatric, vascular, or endocrine surgery, leaving fewer surgeons available for emergency and trauma coverage [10].

These converging trends created a perfect storm that threatened the sustainability of traditional trauma and emergency general surgery models. Trauma call schedules often proved disruptive to maintaining an elective surgical practice, while attempts to establish trauma surgery as a standalone specialty encountered multiple obstacles. The continuous coverage requirements, relatively low revenue generation potential, and diminishing opportunities to maintain surgical skills made a focused trauma practice unattractive to many surgeons. The decreasing need for operative trauma management, resulting from both improved nonoperative approaches and the delegation of many procedures to other specialties like interventional radiology, neurosurgery, and orthopedics, further exacerbated these challenges. Consequently, the traditional trauma and emergency general surgery model became increasingly untenable except perhaps in major urban trauma centers with high volumes of penetrating trauma [11].

In this context, ACS emerged as an innovative solution by combining three critical components: trauma surgery, emergency general surgery, and surgical critical care. This integrated approach addressed multiple systemic issues simultaneously. It ensured reliable 24/7 coverage for time-sensitive surgical emergencies while providing surgeons with a more diverse and sustainable practice mix. By incorporating emergency general surgery cases alongside trauma, ACS maintained surgeons' operative skills and clinical acumen. The inclusion of surgical critical care added another dimension of expertise and created more balanced practice opportunities. Furthermore, this model offered better alignment with modern healthcare delivery systems and reimbursement structures compared to standalone trauma surgery [12].

The ACS model has proven particularly valuable in addressing the growing gap in emergency surgical care availability. It created a new generation of broadly trained acute care surgeons capable of managing the full spectrum of urgent and life-threatening surgical conditions, from traumatic injuries to surgical emergencies and critical care management. This comprehensive approach not only improved patient care but also enhanced career sustainability for surgeons specializing in acute care. Today, ACS stands as a vital specialty that meets the evolving needs of healthcare systems, trauma patients, and surgical professionals alike [12].

As the landscape of ACS evolved, the field shifted from a traditional reliance on surgical interventions to a more holistic, comprehensive approach that integrates advanced critical care strategies. For instance, the COVID-19 pandemic underscored the importance of this transition, as health systems faced immense strain, prompting a reevaluation of priorities and resource allocation. Extracorporeal Membrane Oxygenation (ECMO), once reserved for a narrow subset of patients, became a crucial tool in managing severe respiratory failure, highlighting the role of critical care in ACS. Similarly, debates over the use of invasive tools like pulmonary artery catheters and transfusion strategies reflect a broader movement towards evidence-based, less invasive management [13].

Building on this shift, the field of ACS has increasingly moved "beyond the knife" to incorporate a more comprehensive perioperative and critical care approach. This transition is not just about improving surgical outcomes but also about enhancing the overall management of surgical patients, particularly those with complex, multisystem needs. In a study in Canada, this evolution has been marked by the development of dedicated acute care surgery programs that provide focused care for non-trauma surgical emergencies over defined periods, improving access and outcomes for these patients. As highlighted by Ball et al, the model has been integral in improving patient care by creating structured, well-coordinated services and emphasizing the importance of academic research and specialized fellowship training. The benefits of this system are evident in better resource management, reduced emergency room congestion, and potentially improved patient outcomes. As the field continues to grow, a critical component of its future will be the integration of evidence-based practices to further refine and optimize perioperative care in surgical emergencies [2].

Innovations in acute care surgery

Artificial Intelligence (AI) in Acute Care Surgery and Surgical Critical Care

Artificial intelligence is increasingly being integrated into medical practice; however, its application in emergency care and acute care surgery remains relatively new [14]. Acute care surgeons routinely make real-time, life-saving decisions with both immediate and long-term implications for patient outcomes. These decisions occur in environments characterized by time pressure, limited staffing, overstretched infrastructure, and high cognitive load, all of which increase the risk of clinical errors and adverse events affecting both patients and healthcare workers [15].

AI and Data-Driven Critical Care

Artificial intelligence refers to computer systems capable of reasoning, learning, and adapting based on prior exposure, while machine learning (ML) represents a subset of AI that analyzes large datasets to generate predictive algorithms [16]. In the ICU, ML enables interpretation of high-volume physiological and laboratory data to improve early detection of complications and guide treatment decisions. Poor outcomes in critical care are frequently attributed to variability in clinical practice, data overload, and medication errors [17]. AI-driven clinical decision support (CDS) systems address these issues by integrating multiple data inputs and aligning recommendations with best-practice guidelines [17].

Machine Learning in Early Sepsis Detection and Hemodynamic Monitoring

Early detection and prediction offer the greatest potential impact on outcomes. The Early Mortality Prediction for Intensive Care Unit-Random Forest (EMPICU-RF) model demonstrated superior mortality prediction compared with traditional scoring systems, including the Sequential Organ Failure Assessment (SOFA) and quick SOFA (qSOFA) scores [16]. Machine learning models have been trained to detect respiratory distress, sepsis, and hemodynamic instability [17].

The Artificial Intelligence Sepsis Expert (AISE) model predicts real-time sepsis onset approximately 4-12 hours earlier than conventional detection thresholds [18]. Similarly, the Acumen Hypotension Prediction Index (HPI) forecasts hypotensive episodes before and after cardiopulmonary bypass surgery using invasive and non-invasive arterial pressure waveform data [18].

Predictive Analytics for Post-Surgical Complications

Post-surgical complications affect approximately 10% of surgical patients, leading to increased mortality, prolonged hospital stay, and higher healthcare costs [19]. AI-based models, including recurrent neural network (RNN) variants developed by Chen et al., demonstrate higher predictive accuracy than traditional risk stratification tools [20]. However, major limitations persist. A systematic review using the Prediction Model Risk of Bias Assessment Tool (PROBAST) found that only 13% of 103 AI-based prediction studies underwent external validation, limiting clinical generalizability [21]. Despite this, El Moheb et al. demonstrated that AI outperformed surgeons in predicting mortality, ventilator dependence, hemorrhage, and pneumonia [22].

Madani et al. trained deep neural networks using 308 anonymized laparoscopic cholecystectomy videos from 37 countries, involving 153 surgeons and 136 institutions, with data spanning 2008-2019 [23]. Ten frames per video were analyzed to identify safe (“go zone”) and hazardous (“no-go zone”) dissection areas. The model successfully recognized critical anatomy even in distorted inflammatory or traumatic settings, highlighting AI’s potential to reduce bile duct injuries in acute care surgery [23].

Robotic Surgery in Acute Care

Robotic surgery has been widely adopted in elective surgery for over a decade, but its role in acute care surgery remains limited [24]. Gage et al. described the implementation of a robotic acute care surgery programme using a multidisciplinary approach, robotics-trained staff, dedicated operating rooms, and 24/7 robotic availability [24]. Robotic acute care cases increased from 77 cases in 2022 to 172 cases in 2023, enabling two additional cases per day.

A systematic review by De’Angelis et al. confirmed increasing use of robotic surgery in emergency settings but highlighted challenges including longer operative times, increased costs, and the need for continuous access and patient-selection guidelines [25]. A cohort study by Lunardi et al., analyzing over one million emergency surgical cases (2013-2021), found that robotic surgery was associated with reduced conversion to open surgery, shorter hospital stays, and increased utilization over time [26].

Telemedicine in Acute Care Surgery and Surgical Critical Care

Telemedicine improves rapid diagnosis, decision-making, and access to specialist care, particularly in remote or resource-limited settings [27]. Its use expanded during the COVID-19 pandemic through virtual ICUs, remote monitoring, and triage, reducing clinician exposure and optimising resource use [28]. At Hamad Medical Corporation, telemedicine replaced in-person postoperative outpatient visits for ACS patients, using structured phone follow-ups with selective in-clinic review. This approach resulted in high patient and clinician satisfaction, reduced time and costs, and maintained patient safety as measured by emergency department visits and readmission rates [27].

A 2025 systematic review by Nouh et al. involving 25 studies and 45,097 patients found that telemedicine improved trauma assessment accuracy, increased Injury Severity Score detection, and transfusion rates, but showed neutral effects on mortality and transfer times, supporting its role as an adjunct rather than a replacement for traditional ACS pathways [29].

Enhanced Recovery After Surgery (ERAS) in ACS

ERAS is a multimodal, evidence-based perioperative approach designed to minimize surgical stress, reduce complications, and accelerate recovery [28]. ERAS protocols include pre-, intra-, and postoperative interventions, multimodal analgesia, early nutrition, and early mobilization. Prolonged bed rest is a major contributor to functional decline in hospitalized patients [30], while early mobilization is safe and associated with improved outcomes across multiple randomized controlled trials [30]. ERAS guidelines consistently reduce length of stay and complication rates across surgical specialties [31]. Stavros et al. analyzed data from 629 hospitals and over 1.5 million patients, demonstrating that higher ERAS adherence significantly reduced hospital stay and improved perioperative outcomes [32]. Stumpo et al. reported improved patient satisfaction, reduced pain, and shorter ICU and hospital stays following ERAS implementation after craniotomy [33].

In emergency abdominal surgery, ERAS implementation (n=80) resulted in 50% protocol adherence by postoperative day two, higher laparoscopic rates (40% vs 12%, p=0.002), a three-day reduction in length of stay (9 vs 12 days, p=0.002), and cost savings of €1,022.78 per patient, with similar morbidity and mortality [34]. Purushothaman et al. randomized 60 trauma laparotomy patients and showed ERAS significantly reduced hospital stay (3.3 vs 5.0 days, p<0.01), earlier removal of nasogastric tubes, urinary catheters, and drains, earlier diet initiation, increased use of epidural analgesia (63% vs 30%), NSAIDs (93% vs 67%), and DVT prophylaxis (100% vs 70%), without increased complications or readmissions [35].

Challenges in acute care surgery

End-of-Life Decision-Making and Resource Allocation

End-of-life decisions in ACS are time-sensitive and ethically complex, involving resuscitation, ventilation, artificial nutrition, withdrawal of treatment, and terminal sedation [33]. External pressures may influence intervention decisions even when perceived as futile [36]. Conflicts often arise between patient autonomy and professional responsibility, but can be mitigated through improved communication and realistic expectation-setting [37].

Studies using quality-adjusted life years (QALYs) and NICE end-of-life policies found limited public preference for prioritizing life-extending treatments solely due to terminal status, with decisions driven more by treatment benefit magnitude [38,39]. Similar findings were reported in the Netherlands [39]. Home-based palliative care models reduce unnecessary emergency visits, ICU admissions, and futile surgery, alleviating moral distress and improving patient dignity [40].

Acute Care Surgeon Shortage and Burnout

Critical care professionals report burnout rates exceeding 50%, driven by high mortality exposure, ethical stress, staffing shortages, and workload intensity [41-43]. ACS surgeons experience heavier workloads than other specialties: 74% work >60 hours/week, 15% >81 hours/week, compared with colorectal (57 hours), vascular (61 hours), and orthopedic surgeons (52 hours) [44-46]. Nearly 50% of ACS work occurs at night and weekends [43].

A 2022 AAST survey found 92% of ACS surgeons felt they worked harder than peers, and 96% believed excessive hours threatened workforce stability [46]. Burnout prevalence of 74% has been reported, driven by staffing deficits, inefficient workflows, and organizational culture [47]. A longitudinal intervention replacing 24-hour calls with 12-hour shifts and protected academic time significantly improved burnout metrics without reducing productivity [43].

Lack of Financing

Despite evidence of financial viability, ACS programmes face chronic underfunding due to misconceptions, high fixed costs, and unfavourable reimbursement structures [46,48]. Unlike elective specialties, ACS lacks dedicated funding streams, despite enabling downstream revenue for orthopedics, neurosurgery, transplantation, and rehabilitation [49]. Sustainable financing requires improved cost-accounting, policy reform, and trauma centre designation funding [49].

Global gaps in ACS

ACS adoption varies widely worldwide, with limited implementation in low- and middle-income countries due to infrastructure and resource constraints [50]. While the ACS model is well established in North America and parts of Europe, adoption remains inconsistent across Africa, Asia, and South America [3,50]. Models differ significantly in staffing, operating room access, trauma integration, and critical care involvement across regions [48,51-63].

Future of ACS

The future of ACS will be driven by artificial intelligence, robotics, telemedicine, simulation-based training, and global collaboration. Evidence consistently demonstrates improved clinical outcomes and economic benefits associated with dedicated ACS teams, reinforcing their essential role in modern healthcare systems [3,8,64-69].

## Conclusions

ACS has fundamentally evolved into a vital, comprehensive specialty that moves "beyond the knife" to integrate critical care and innovative technologies into the management of surgical emergencies. The evidence demonstrates that dedicated ACS models significantly improve patient outcomes, reduce complications and hospital length of stay, and can be economically viable. However, the field faces profound challenges, including high rates of surgeon burnout due to excessive workloads, complex ethical decisions, and persistent underfunding. Moreover, the global implementation of ACS is highly heterogeneous, with critical gaps in low- and middle-income countries. The future of ACS depends on a multi-faceted approach: leveraging AI, robotics, and telemedicine to enhance care; implementing systemic changes to support the workforce; and pursuing global initiatives to standardize and fund equitable emergency surgical services. Ultimately, strengthening ACS systems is essential for improving survival and recovery for patients facing time-sensitive surgical crises.
